# Assessing the social vulnerability to malaria in Rwanda

**DOI:** 10.1186/1475-2875-14-2

**Published:** 2015-01-07

**Authors:** Jean-Pierre Bizimana, Emmanuel Twarabamenye, Stefan Kienberger

**Affiliations:** College of Science and Technology, Geography Department, University of Rwanda, PO Box 212, Butare, Rwanda; Interfaculty Department of Geoinformatics - Z_GIS, University of Salzburg, Schillerstrasse, 305020 Salzburg, Austria

**Keywords:** Malaria, Social vulnerability, Composite indicator, Sensitivity analysis, Rwanda

## Abstract

**Background:**

Since 2004, malaria interventions in Rwanda have resulted in substantial decline of malaria incidence. However, this achievement is fragile as potentials for local malaria transmissions remain. The risk of getting malaria infection is partially explained by social conditions of vulnerable populations. Since vulnerability to malaria is both influenced by social and environmental factors, its complexity cannot be measured by a single value. The aim of this paper is, therefore, to apply a composite indicator approach for assessing social vulnerability to malaria in Rwanda. This assessment informs the decision-makers in targeting malaria interventions and allocating limited resources to reduce malaria burden in Rwanda.

**Methods:**

A literature review was used to conceptualize the social vulnerability to malaria and to select the appropriate vulnerability indicators. Indicators used in the index creation were classified into susceptibility and lack of resilience vulnerability domains. The main steps followed include selection of indicators and datasets, imputation of missing values, descriptive statistics, normalization and weighting of indicators, local sensitivity analysis and indicators aggregation. Correlation analysis helped to empirically evidence the association between the indicators and malaria incidence.

**Results:**

The high values of social vulnerability to malaria are found in Gicumbi, Rusizi, Nyaruguru and Gisagara, and low values in Muhanga, Nyarugenge, Kicukiro and Nyanza. The most influential susceptibility indicators to increase malaria are population change (r = 0.729), average number of persons per bedroom (r = 0.531), number of households affected by droughts and famines (r = 0.591), and area used for irrigation (r = 0.611). The bed net ownership (r = −0.398) and poor housing wall materials (0.378) are the lack of resilience indicators that significantly correlate with malaria incidence.

**Conclusions:**

The developed composite index social vulnerability to malaria indicates which indicators need to be addressed and in which districts. The results from this study are salient for public health policy- and decision makers in malaria control in Rwanda and timely support the national integrated malaria initiative. Future research development should focus on spatial explicit vulnerability assessment by combining environmental and social drivers to achieve an integrated and complete assessment of vulnerability to malaria.

## Background

In Rwanda, malaria is mesoendemic in the lowlands and hypo-endemic in highlands. Malaria is a public health concern in Rwanda because the entire population of is at risk for malaria [1], including an estimated 2.2 million children under five years of age and 443,000 pregnant women [2]. Nearly 63% of the country is epidemic-prone, while the remaining of the country is characterized by a stable and endemic malaria transmission. Malaria transmission occurs throughout the year with two peaks in rainy seasons. In addition to climate suitability, other factors that influence malaria transmission, including human settlements near the marshlands, internal population movement and migrations, cross-border movement of people and irrigation schemes [3].

In 2005, the Government of Rwanda benefited from US Presidential Malaria Initiative (PMI) to reduce malaria-related deaths in Africa. In Rwanda, PMI is expanding coverage of insecticide-treated mosquito nets (ITNs), indoor residual spraying (IRS) with insecticides, and prompt use of artemisinin-based combination therapy (ACT) for malaria treatment [1]. These interventions resulted in substantial decline in malaria transmission. For example, after falling down between 2006 and 2008 owning to an increase in ITNs coverage, malaria increased again 2009 because of limited coverage with ITNs. The number of malaria cases declined again in 2010 following an ITN distribution campaign [4]. This achievement is, however, fragile as potentials for local malaria transmission remain. The entire population is at risk particularly children aged under five years and pregnant women [5]. Highland communities suffer from epidemic malaria attributed to low immunity [6]. Despite the decreasing malaria through intensified interventions in Rwanda, the environmental changes induced by high population pressure might expose the highland populations to an increase risk of malaria and its epidemic particularly if the current interventions are not sustained [7].

A study in the southern Rwanda highlands revealed that asymptomatic malaria was associated with low social conditions and ineffective use of bed nets in local communities [8]. Failure to sustain malaria control and reduction in bed nets use may result in malaria resurgence in Rwanda [9]. For instance, an upsurge of malaria incidence in 2009 was linked to a short delay in bed net provision [10]. This demonstrates the fragility of progress made and the need to maintain malaria control despite decreasing malaria in Rwanda [9].

The declining malaria provides new challenges for its elimination, including for example the resistance of malaria vectors to insecticides and livelihood activities that increase exposure to mosquito bites [11]. With intensive use of bed nets, immunity to malaria would develop more slowly under the reduced transmission, leading to a longer period of susceptibility [12]. A declining malaria transmission in Rwanda may therefore impair the development of immunity, which would increase the populations vulnerability to severe malaria if the control measures are not maintained [13]. Because of population movements and migrations, malaria is imported from endemic areas to low-transmission areas. Land use changes are clustering malaria where populations share the same social and environmental factors [14], such as high population densities and pressure associated with land use changes and economic activities which increase mosquito breeding sites [15]. Therefore, approaches to malaria reduction need to be aligned with these changes by adopting new strategies [16].

Recently, an integrated vector management strategy was adopted in Rwanda as a framework for interventions based on local ecology, malaria epidemiology and social factors [1]. IVM strategy targets multiple vectors and different ecological and socio-economic settings. To be effective, IVM strategy should encompass environmental modifications through infrastructural development and sanitation services to regulate not only the vectors, but also the mosquito biting exposure. Additionally, IVM should improve public health and quality of life while minimizing the social disparities [17]. The spatial assessment of social vulnerability to malaria is, therefore, a well-timed support to the IVM initiative related to public health improvement and social disparities among the populations at risk of malaria infection.

In any society, there are groups of people who have limited control over their ability to admit to illness, mobilize resources, access health facilities and services, and make decisions. This lack of personal control places them in a position in which they can be considered to be socially vulnerable to disease infection [18]. Social vulnerability assessment in the arena of vector-borne diseases highlights the importance of social factors that make some groups or individuals more susceptible to infection and more limited in their ability to respond to illness than others [19]. Until now, the public health approach to malaria in sub-Saharan Africa (including Rwanda) is based on Global Health Initiative strategy, which concentrates on reducing malaria burden by funding specific interventions to strengthening the health care system [1]. Although these programmes have temporarily reduced the overall malaria infection rates countrywide, they are unlikely to be effective in producing sustainable reductions of malaria without addressing the proximate causes of malaria transmission and ultimate or efficient causes of malaria incidence in social structure, agro-ecological settings and demographic pressure of the country [20]. In addition, if the efforts to reduce malaria are solely concentrated on health care sector and malaria disease control, this may fail to address other factors that shape the health of individuals or community, such as rural housing, food security and employment [21]. The reduction of malaria burden requires, therefore, an integrated design of interventions that are placed within the broader social context of malaria transmission and incidence.

While the vulnerability to malaria is a multidimensional concept encompassing both environmental and social factors, its complexity cannot be measured by a single indicator value. The aim of this paper is to apply an integrated composite indicator approach for assessing the social vulnerability to malaria in Rwanda, independent of the spatial distribution of malaria based on a purely statistical approach and a composite indicator approach. Contextually, the social vulnerability to malaria encompasses a broader social context of malaria incidence in addition to environmental factors of malaria transmission. It reflects the predisposition of the populations or individuals to malaria infection and theirs ability to mitigate the risk of malaria. The results from this study indicate the most vulnerable districts in Rwanda as a combination of susceptibility to not withstand malaria infection and lack of resilience to anticipate, to cope with or to recover from malaria episodes in Rwanda.

The social vulnerability to malaria in Rwanda is timely supporting the national integrated malaria initiatives, which seek to improve the efficacy, effectiveness and sustainability of malaria control interventions through the inter-sectors collaboration, resources allocation and health infrastructure development [1]. This assessment is also intending to provide information on vulnerability indicators that should be given priority in targeting intervention strategies and allocating limited resources in order to reduce the existing susceptibilities, strengthen community resilience, and thus reduce malaria burden in Rwanda.

## Methods

### Conceptual setting

Vulnerability is a well-documented concept in the fields of disaster risk reduction and climate change adaptation [22, 23]. Vulnerability to natural hazards refers to the conditions determined by physical, social, economic, and environmental factors that increase the susceptibility of a community to hazards [24]. Füssel [25] stressed the need of an integrated approach for climate change vulnerability assessment which would also consider non-climatic factors. In line with an integrative approach towards vulnerability assessments, the European-funded research project MOVE (Methods for the Improvement of Vulnerability Assessment in Europe) identified key factors and dimensions that need to be addressed [23]. The MOVE vulnerability framework considers society and environment as a coupled system between environment, hazard and society. The core elements in MOVE framework are exposure, susceptibility and lack of resilience. Vulnerability is further characterized by different scales (local, regional, national) and dimensions (environmental, ecological, social, economic, institutional, cultural) [26].

While the MOVE framework is oriented towards natural hazards and climate change adaptation, some common features can emerge when assessing the social vulnerability to vector-borne diseases like malaria. Recently, there has been growing recognition that social and cultural factors significantly influence the distribution of health and illness, and that issues of inequity affect how disease incidences are distributed and treated [27]. From an international perspective, public health community involved in malaria reduction is also increasingly receptive to broader and more encompassing definitions of the malaria problem. The biomedical community is expanding its vision on malaria because existing tools of known efficacy to anticipate mosquito bites and cope with malaria infection may be of limited value because of social barriers to their effective implementation [28]. Moreover, ignoring the social determinants of malaria is likely to allow public decision- and policy-makers to concentrate on malaria mosquitoes and not to be concerned with thorny problems of poverty and inequalities in health care facilities, access to health treatment, distribution of land and capital resources that play also a key role in malaria incidence [21, 29]. Packard presented the similar argument when highlighting that it is an unlikely possibility to eradicate malaria by only distributing mosquito nets, while sanitation is not ensured. The burden of malaria cannot be reduced by simply attacking anopheline mosquitoes and malaria parasites. Rather, removing the barriers that prevent people from achieving and maintaining health is also an imperative task that can help to combat malaria from multiple fronts [20]. Consequently, there is a need to move away from a narrow biomedical approach which viewed malaria as a problem of mosquitoes, parasites and vector control activities toward a more integrated perspective which is tied to social conditions that increase the rates of malaria morbidity and mortality [29].

As pointed out by Ribera and Hausmann-Muela, malaria should not be seen as single illness episodes, but as successive sequences of interconnected events that, in a context of poverty and social inequalities, determine the course of the illness as well as the health-seeking process [30]. Similarly, Stratton and colleagues advocated for an action paradigm that links the traditional proximal arenas of interventions with malaria’s fundamental causes by addressing the environmental, economic, and political dimensions of malaria risk. They concluded that strategies that exclusively focus on reducing exposure to mosquitoes or treating malaria may provide tangible health benefits, but their outcome will be less effective in the long run at reducing total health burden than approaches aimed at underlying causes of differential vulnerability given the high poverty rates in most malaria endemic regions [31]. This was also supported by Jones and Williams, when advocating for a comprehensive approach for diseases control. In their discussion on social burden of malaria, they urge for taking a broader social perspective to malaria burden and advocate for a shift to integrated designs of interventions that are placed within the broader social, cultural, political and economic context [18].

**Figure 1:**
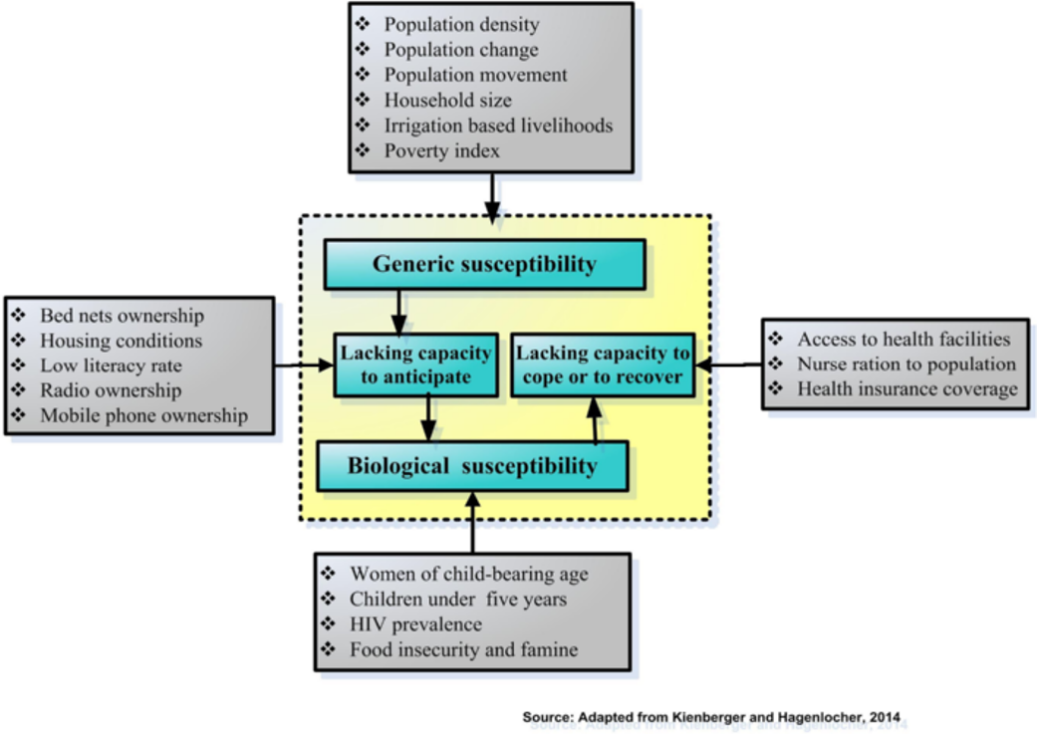
**Adapted framework of social vulnerability to malaria.**

In the assessment of vulnerability to diseases, there is a growing literature which defines the vulnerability to malaria based on biological and disease-related factors, such as age, pregnancy and immunity [32–34]. Other studies go beyond individual behaviour or characteristics, to larger-scale social and economic conditions, because some societies have been more successful in addressing malaria disease than others [35]. In line with this integrative assessment of social vulnerability to malaria, this study is drawn on a holistic risk and vulnerability framework which was developed by Kienberger and Hagenlocher in the context of vulnerability to vector-borne diseases [36]. Figure 1 shows the adopted and applied framework of the domains of social vulnerability to malaria and examples of relevant indicators.

Based on recent publications by Kienberger and Hagenlocher [36] and Hagenlocher *et al.* [37], the vulnerability to malaria is defined as the predisposition of the population to malaria burden. It is characterized by different interrelated domains, generic and biological susceptibility, and lack of resilience. The distinction between generic and biological factors pre-disposing a community to malaria burden versus those that determine its ability to anticipate responding to, to cope with or to recover from malaria burden should be relevant for decision makers [36, 38].

Vulnerability assessments often employ indicators to better reflect complexity and real-world information into a format that is relevant for decision-making. Despite their alleged weaknesses, composite indicators provide an essential tool at the science–policy interface [39]. The use of composite indicators is an important way to communicate and monitor the vulnerability, and allows comparisons to be made across different geographical areas [40]. By combining social and environmental indicators, geographic variations of vulnerability to malaria can be illustrated. The vulnerability map can, therefore, indicate the relative levels of vulnerability, where there is uneven capacity for preparedness and response, and where resources might be used effectively to reduce prevailing vulnerability. This provides information to decision-makers for targeting vulnerable populations while improving their resilience [41]. While recognizing that vulnerability is a multidimensional and dynamic condition at different spatial and temporal scales, there is a need to assess how social drivers influence the vulnerability to malaria among the districts of Rwanda using a composite indicator approach.

### Selection and justification of indicators

One of the key parts of this study is the selection of relevant indicators for assessing the social vulnerability to malaria. Guided by the vulnerability framework, 19 indicators have been identified. Table 1 shows vulnerability domains, indicators used and their corresponding weight from principal component analysis (PCA) and information on sources of datasets as well.

**Table 1:** **Malaria vulnerability indicators**

Domain	Sub-domains	Indicators	Proxies	Sign	Source	Weights
**Susceptibility**	Generic susceptibility	Population pressure	Population density in sq km	+	NISR 2012	0.087
			Population change 2002-2012	+	NISR, 2012	0.056
		Population movements	Number of arriving populations	+	EICV3 2011	0.126
		Households size	Average number of persons per bedroom	+	EICV3 2011	0.050
		Livelihoods	Land area used for irrigation	+	EICV3 2011	0.081
		Poverty index	Number of poor populations	+	DHS 2010	0.134
	Biological susceptibility	Pregnancy	Women of child-bearing age	+	NISR 2012	0.110
		Age	Number of children under five years	+	NISR, 2012	0.113
			Number of population above 65 years	-	NISR, 2012	0.060
		HIV	HIV prevalence in adults aged 15-49	+	DHS2010	0.120
		Malnutrition	% of households affected by drought and famines	+	EICV3 2011	0.064
**Lack of resilience**	Capacity to anticipate mosquito biting exposure	Education level	Low literacy rate	+	DHS, 2010	0.133
		Housing condition	Number of households in poor housing wall materials	+	DHS2010	0.162
			Number of households in poor housing roof materials	+	DHS2010	0.200
		Access to media	Households without radio	+	DHS2010	0.113
			Households without mobile phone	+	DHS2010	0.127
		Protection measures	Number of households without bed nets	+	DHS, 2010	0.113
	Capacity to cope/recover	Access to health services	Number of health facilities	-	MoH	0.086
			Nurse ratio to population	+	MoH	0.085

The positive sign indicates if the high indicator values increase the vulnerability while the negative sign indicates the high indicator values decrease the vulnerability. Weights were derived for the individual indicators using the principal component analysis. NISR = National Institute of Statistics of Rwanda; EICV = Integrated Household Living Conditions Survey; DHS = Demographic and Health Survey; MoH: Ministry of Health.

Generally, high density and population pressure in highlands results in limited land resources and increases human susceptibility to diseases [42]. This is relevant for Rwanda highlands, where demographic pressure has significantly modified the local environment during the past decades [15]. Swamps were reclaimed by agriculture to feed an ever-increasing population. Water requirements for irrigation have led to modifications of surface waters [43]. Since malaria incidences are rooted in livelihood activities which interplay with the ineffective use and non-use of bed nets [44], irrigation-based agricultural practices may increase the susceptibility to malaria infection [45]. These environmental changes are likely to influence malaria incidence if adequate protection measures are not implemented in nearby communities [46]. In additionally, high population density and pressure have significantly influenced the environmental degradation and declining landholdings [47, 48], which have pushed people to settle near unsuitable sites with more exposure to mosquito bites [15].

Populations with little immunity may move into malaria-prone areas where they are more vulnerable [49]. The Rwandan population increased in past decades from approximately 2.6 million in 1960 to 8.2 million in 2002 and 10.6 millions in 2012 [50]. This was accompanied by environmental degradation and decreasing landholding size, pushing people to settle near unsuitable sites with more exposure to mosquito bites [15]. The resettlement of non-immune people in endemic zones was accompanied by sporadic malaria epidemics [51].

The household size is often associated with malaria incidence [52]. The more people are sleeping together in the same room, the higher the probability of spreading infection to a new person. Below a certain threshold number of persons sleeping together, infection rates drop below the replacement rate and disappear even without other control measures. The threshold is likely crossed when the average household size drops below four persons [53]. Moreover, the increasing number of persons per room results in improper use of bed nets and increased probability of being infected by malaria parasites.

From biological perspective, the susceptibility to malaria reflects the efficiency with which an infective mosquito infects humans. It is a function of immunity which depends on age, pregnancy or co-infection with other diseases [32]. Pregnant women and children under five years of age are more susceptible to severe malaria since women’s immunity is temporarily reduced during pregnancy, while the immune system of small children is not fully developed [54]. When HIV and malaria co-infect, a severe malaria should be expected [55]. In non-pregnant women, HIV was found to roughly double the risk of malaria by impairing the immune response and decreasing the ability to withstand malaria infection [56], and reducing the efficacy of drugs [57]. A recent study in Rwanda confirmed higher malaria prevalence among HIV-positive, pregnant women [58]. With regard to age, adults would be able to withstand malaria infection because of acquired immunity from previous exposure to mosquito bites [59]. Famines and food shortage induced by drought also result in population movements and migration, increasing exposure to malaria in endemic lowlands, thus rising the malaria incidence in returning population to the highlands [60]. Recurrent drought in Rwanda caused crop failures and food shortages, then threatening the most vulnerable populations with malnutrition and famine, especially in the Eastern Province of Rwanda [61].

The lack of resilience relates to the capacity to anticipate mosquito-biting exposure and to limited access to health infrastructure and means to recover from malaria episodes. This capacity may be influenced by the level of education, awareness about malaria transmission and prevention, and access to protection measures. The coping capacity relates to access to health care services and to adequate and effective treatment [35]. In this study, this encompasses protection measures, housing conditions, education level and improved knowledge about malaria which lead to better use of malaria interventions [62]. The use of bed nets is the most accepted protection method against malaria. For bed nets to be effective, coverage must be high, bed nets should be retreated promptly and individuals should properly deploy their bed nets each night. The more households own and use bed nets, the greater the benefit to neighbouring households without bed nets [63]. Indoor residual spraying (IRS) application on houses’ walls and roofs and on domestic animal shelters kills the adult mosquitoes that rest on these surfaces. It reduces the longevity and density of mosquitoes so that they can no longer transmit malaria parasites [64]. Information on IRS was not however factored in this study because the related data was not available for the entire study area. Once this information is available for the entire country, this could additionally reduce uncertainties in vulnerability assessment.

Poor housing quality provides less protection against mosquitoes [65]. Houses with more malaria-infected children are more likely to have mud walls, open eaves and absent ceilings than those with uninfected children [66]. Despite the significant reductions in malaria transmission by high coverage of bed net use in Rwanda, high numbers of host-seeking malaria vectors in rural areas may be found indoors due to poor housing quality. Education levels may influence malaria incidence since it affects the knowledge about malaria prevention and control. Over recent years there has been emphasis on that level of education and improved knowledge about malaria leads to better use of malaria interventions [62]. Nevertheless, association between malaria and education may be due to its role as a proxy for poverty. Media communication plays a strong role in malaria control to ensure that bed nets are used appropriately [67]. Mobile phone technology is also an efficient method for rapidly detecting malaria patients and reduce malaria deaths in remote rural areas [68].

In terms of coping and recovering capacity, the vulnerability to malaria may be influenced by access to treatment and prompt access to effective malaria treatment is central to the success of malaria control worldwide. The Roll Back Malaria partnership has set for 2010 a target of ensuring that 80 percent of those suffering from malaria have prompt access to, and are able to correctly use, affordable and appropriate treatment within 24 hours of symptoms onset [69]. A strategy to provide such access should take into account poor populations in malaria-endemic zones who are particularly inadequately served by the health system [70]. In such areas, convergence of malaria prevalence and poor health care infrastructure can result in high malaria incidence rates. Moreover, primary care facilities are increasingly becoming the focal point for distribution of intervention strategies, but physical access to these health facilities may limit the extent to which communities can be reached [71].

### Construction of a composite index of social vulnerability to malaria

**Figure 2:**
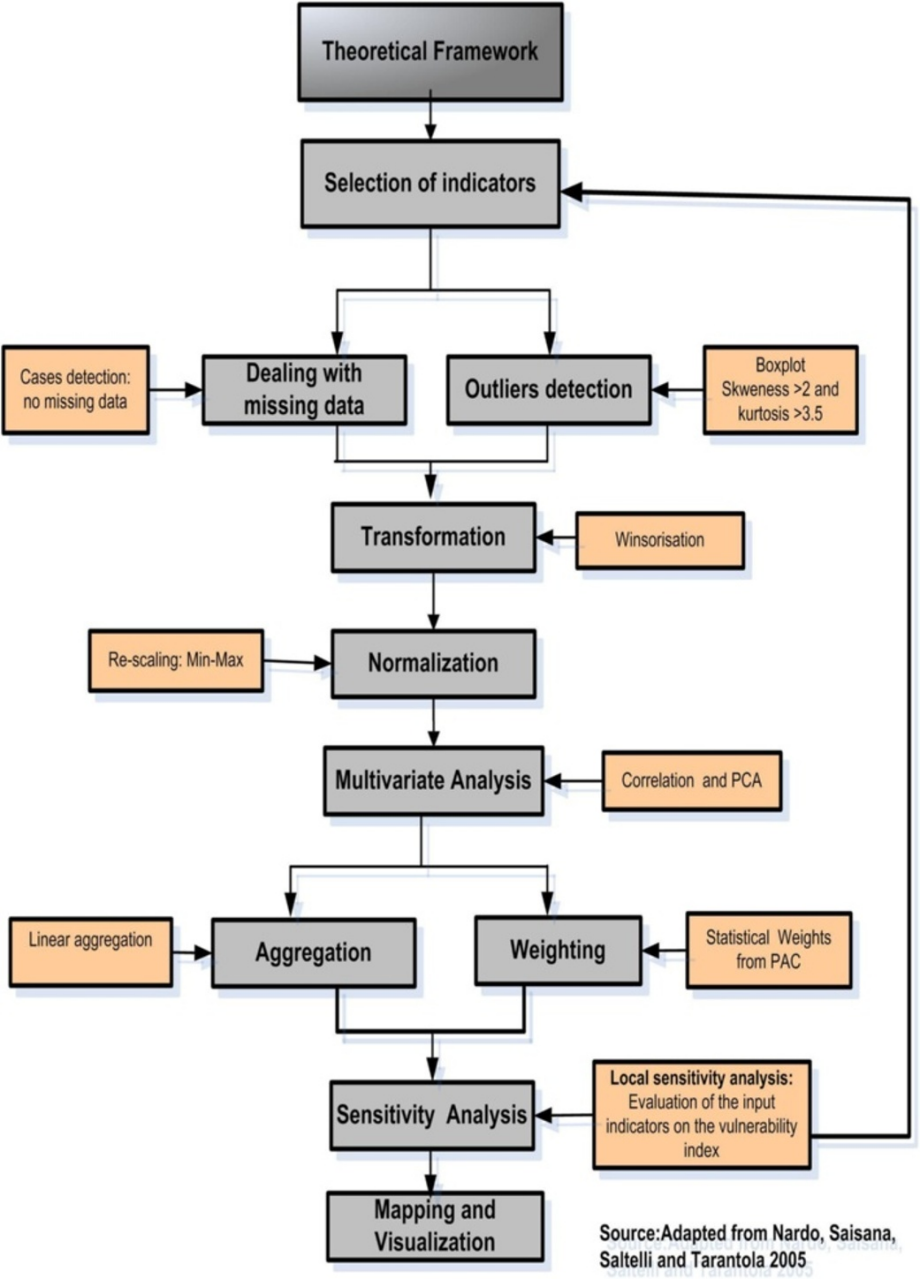
**Steps in constructing a composite vulnerability indicator.**

To construct a composite index of social vulnerability, a methodology developed by Nardo *et al.* [72] was adopted. The steps followed were: the selection of relevant indicators, identification of appropriate datasets, imputation of missing data, and descriptive statistics for data exploration (distribution and multicollinearity among indicators, outliers’ detection), data transformation and normalization. The PCA was performed to weight indicators for further aggregation. Prior to aggregation and final vulnerability index creation, the local sensitivity analysis was applied to evaluate the influence of indicators on final index. Then Spearman correlation the regression analysis was applied to provide evidence of association between the used indicator and malaria at district level by highlighting the most sensitive indicators. A final composite index of vulnerability was then computed and visualized for each district. Figure 2 illustrates the main steps followed in constructing a composite index of social vulnerability to malaria.

Descriptive statistics were used for each indicator to evaluate the degree of missing data and potential outliers. Skewness with absolute values above 0.2 were taken as highest threshold for multicollinearity to avoid double counting among indicators [73]. Indicators with kurtosis values greater than 3.5 were treated for asymmetric distribution [74]. Outliers were removed by changing outliers’ values to next highest/lowest non-outlier number [75]. Afterwards, outlier analysis was re-run to check if all outliers were eliminated [76].

Since indicators were at different measurement scales, standardization was required before their aggregation [77]. Sometimes high ‘raw’ indicator data values mean low vulnerability, so therefore theses indicators have been transformed to have high values for high vulnerability. The minimum-maximum transformation method was used by accounting for the direction of indicators [78] and using the flowing formula:
1

Where *Vi* is equal to the standardized indicator *i*; *Xi* represents the indicator value before its transformation; *Xi, min* is the minimum score of indicator *i* before its transformation*;* and *Xi, max* as maximum score of indicator i before its transformation. All indicator values were transformed into a relative score ranging from 0 to 1, where higher values imply high vulnerability [74]. A positive sign implies that high indicator values increase the vulnerability (+), while low values decrease the vulnerability and *vice versa*.

Prior to PCA, the correlation coefficient matrix of two vulnerability domain indicators was scanned to check for the values greater than 0.92 as an indication of collinearity as an indication of collinearity [79]. After scanning the correlation coefficient matrix, no value greater than this threshold was found. Since many correlation coefficient values were above 0.300, the PCA would yield the acceptable results [80]. The Kaiser-Meyer-Olkin (KMO) criteria and Bartlett’s test were also performed to examine the data suitability for PCA. The general rule is that the overall KMO should be 0.60 or higher to proceed with PCA. The high values for overall KMO for both susceptibility and lack of resilience indicators were 0.655 and 0.615, respectively, meaning that they are acceptable for PCA.

### PCA for weighting indicators

A PCA was used to assign the weights to individual indicators based on common variance explained. Using a varimax orthogonal rotation, components with eigenvalues larger than one; which contribute individually to overall variance by more than 10%; and cumulatively to more than 60% were chosen [81]. Table 2 illustrates the two extracted components explain 64.54% of total variance for lack of resilience indicators.

**Table 2:** **Variance explained for principal component analysis of lack of resilience indicators**

Component	Initial eigenvalues			Rotation sums of squared loadings		
	Total	% variance	Cumulative %	Total	% variance	Cumulative %
1	3.501	43.767	43.767	2.732	34.15	34.15
2	1.662	20.779	64.546	2.432	30.396	64.54
3	0.917	11.463	76.009			
4	0.759	9.491	85.500			
5	0.507	6.339	91.839			
6	0.420	5.254	97.092			
7	0.175	2.184	99.276			
8	0.058	0.724	100			

As earlier mentioned, components with eigenvalues larger than one, individually contributing to overall variance by more than 10%, and cumulatively by more than 60%, were chosen to weight the individual of indicators. Table 3 shows the highest indicator scores in each component and their weights for each single indicator are highlighted in bold.

**Table 3:** **Squared loadings after rotation for lack of resilience indicators**

Lack of resilience indicators	Components		Weights	Scaled weights
	1	2		
Poor housing roof materials	**0.808**	0.000	0.428	0.1999
Poor housing walls materials	**0.656**	0.043	0.347	0.1623
Low literacy rate	**0.536**	0.104	0.283	0.1325
Nurse ratio to populations	**0.343**	0.193	0.182	0.0849
Households without mobile phone	0.209	**0.634**	0.272	0.1274
Households without radio	0.167	**0.563**	0.242	0.1131
Households without bed nets	0.011	**0.466**	0.201	0.0938
Number of health facilities	0.003	**0.428**	0.184	0.0860
*Sums of squared loadings (VE)*	*2.733*	*2.430*	*2.139*	*1.000*
*Total variance*	*5.164*			
*VE/Total variance*	*0.529*	*0.471*		

The highest squared loadings for lack of resilience indicators in each principal component are high highlighted in bold.

The lack of resilience indicators that have the highest squared loading factors in the first principal component are poor housing roof materials, poor housing wall materials, low literacy rate and nurse ratio to populations. The second principal component is heavily loaded by the households without mobile phone, the households without radio, households without bed nets and number of health facilities.

With regard to the susceptibility indicators, three extracted components were able to explain 73.04% of the total variance as shown in Table 4.

**Table 4:** **Variance explained for principal component analysis of susceptibility indicators**

Components	Initial eigenvalues			Rotation sums of squared loadings		
	Total	% variance	Cumulative %	Total	% variance	Cumulative %
1	3.583	32.569	32.569	3.313	30.114	30.114
2	3.202	29.113	61.682	2.877	26.156	56.270
3	1.250	11.363	73.045	1.845	16.775	73.045
4	0.853	7.754	80.799			
5	0.712	6.469	87.268			
6	0.457	4.155	91.423			
7	0.321	2.917	94.340			
8	0.238	2.168	96.508			
9	0.179	1.628	98.136			
10	0.110	0.996	99.132			
11	0.095	0.868	100.000			

The susceptibility indicators with the highest indicator scores in each principal component are highlighted in bold as illustrated in Table 5.

**Table 5:** **Squared loadings after rotation of components for susceptibility indicators**

Susceptibility indicators	Component			Weights	Scaled weights
	1	2	3		
Number of poor populations	**0.776**	0.000	0.031	0.320	0.134
Number of arriving populations	**0.732**	0.001	0.014	0.301	0.126
HIV prevalence in population of 15–49 years	**0.695**	0.000	0.012	0.286	0.120
Population density	**0.505**	0.024	0.158	0.208	0.087
Children under five years of age	0.072	**0.756**	0.003	0.271	0.113
Women of child-bearing age	0.000	**0.735**	0.005	0.263	0.110
Households affected by droughts and famine	0.151	**0.427**	0.012	0.153	0.064
Population above 65 years	0.229	**0.400**	0.149	0.143	0.060
Average number of persons per bedroom	0.063	**0.337**	0.034	0.121	0.050
Land area used for irrigation	0.024	0.003	**0.842**	0.194	0.081
Population change 2002-2012	0.067	0.194	**0.585**	0.134	0.056
**Sums of squared loadings (VE)**	**3.313**	**2.877**	**1.845**	**2.394**	**1.000**
**Total variance**	8.035				
**VE/Total variance**	0.412	0.358	0.230		

The highest squared loadings for susceptibility indicators in each principal component are high highlighted in bold.

The susceptibility indicators that have the highest squared loadings in the first principal component are the number of poor populations, number of arriving populations, HIV prevalence, and population density. Apart from HIV prevalence, most of the highest scored indicators in first principal component are related to generic susceptibility sub-domain. With the exception of the average number of persons per bedroom, the majority of the biological susceptibility sub-domain (children under five years of age, women of child-bearing age, households affected by droughts and famines and elder population above 65 years) dominate the second principal component. The land area used for irrigation and the population change between 2002 and 2012 have an excessive influence in the last principal component.

### Aggregating indicators

Approaches for indicator weighting and aggregation have been subject to debate, but so far no approach is without limitations [82]. Some approaches employ equal weights which ensure transparency and are straightforward, but have been criticized for assignment of implicit equal weights [72]. A study by Hagenlocher *et al*. [37] for modeling the vulnerability to disease based on administrative boundaries concluded expert-based and purely statistical-based modeling approaches reveal similar outputs, indicating that in the absence of local expertise, statistical approaches could be used. The PCA was therefore used since it has the advantage of determining weights which explains the largest variation in original indicators [83]. The weighted sum algorithm was chosen as the most widespread aggregation method. The normalized indicators were first aggregated according to their respective domains. The normalized weighted indicators for each domain were summed using the following formula:
2

SUS and LoR represent the value of vulnerability for susceptibility and lack of resilience domains respectively; W refers to the weight of a single indicator in each domain; and I is the normalized value of the indicator*.* The final composite index was calculated by aggregating the two domains and taking into account the number of indicators in each domain so that the domains grouping the larger number of indicators will have higher weight as follow:
3

Where **n** represents the number of indicators for a given domain; **d** refers to the value of each vulnerability domain while **N** is equal to the total number of indicators. From nineteen indicators that have been identified from literature, eleven indicators were assigned to susceptibility domain, and eight indicators to the lack of resilience domain. For easy visualization of the results, the final index values were normalized within a new range from zero to one, where zero reflects a very low and one a very high social vulnerability to malaria. The higher the values of the vulnerability index, the more the district is vulnerable.

### Sensitivity analysis

Sensitivity analysis evaluates the contribution of individual sources of the uncertainty to the output. For this study, spearman correlation analysis was first used to validate the appropriateness of using indicators using International Business Machines Corporation SPSS statistics 22.0 for windows software. The general aim was to provide empirical evidence of association between indicators and malaria incidence by highlighting the most appropriate to influence malaria incidence at district level. In addition to correlation analysis, local sensitivity analysis helped to assess the influence of the input vulnerability indicators on the vulnerability index. This was achieved by targeting one construction stage at a time, while all other stages are held constant [84]. Consequently, the use of box plots helped to assess the influence of the input vulnerability indicators by discarding one input at the time while keeping all other setting (normalization, weighting and aggregation) equal [85]. This resulted in a series of alternative vulnerability indices. For each district, the alternative index was compared with the reference vulnerability index that takes into account the susceptibility and lack of resilience indicators. The results are displayed in the box plots showing the interquartile range, the minimum and maximum values [36]. The larger the interquartile range, the higher is the influence of the respective input indicator [85].

### Visualization of the results

Because public health decision-makers need the information on the most vulnerable districts and the social drivers of the vulnerability to malaria, a cartographic visualization method was adopted to translate the vulnerability index into a geographic map. ESRI ArcGIS10.2 software was used to display and map the final index of social vulnerability to malaria for each district. To ease interpretation of the assessment results, the final vulnerability index values were normalized within the zero to one range, where zero reflects a very low and one a very high social vulnerability to malaria. The values of the vulnerability index reflect the relative levels of social vulnerability among the district of Rwanda, which means that an index value of 0.00 does not imply the absence of vulnerability. The developed composite index shows the most vulnerable districts to integrated social indicators in terms of susceptibility to not withstand malaria and lack of resilience to anticipate, to cope with or to recover from malaria infection in Rwanda.

## Results

### Vulnerability to malaria and underlying factors

**Figure 3:**
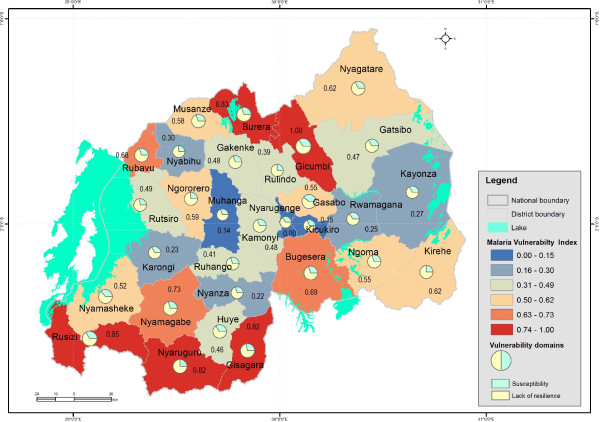
**Levels of malaria vulnerability at district level in Rwanda.**

As shown in Figure 3, high values of vulnerability index to malaria are found in Gicumbi (1.00), Rusizi (0.81), Nyaruguru (0.79), Gisagara (0.71), and Burera (0.67), and districts, and low values in Muhanga (0.00), Nyarugenge (0.10), Kicukiro (0.13), and Nyanza (0.17).

It is very important know that the vulnerability index values reflect the relative levels of vulnerability, which means that the value of 0.00 does not mean the absence of vulnerability to malaria within the district.

**Figure 4:**
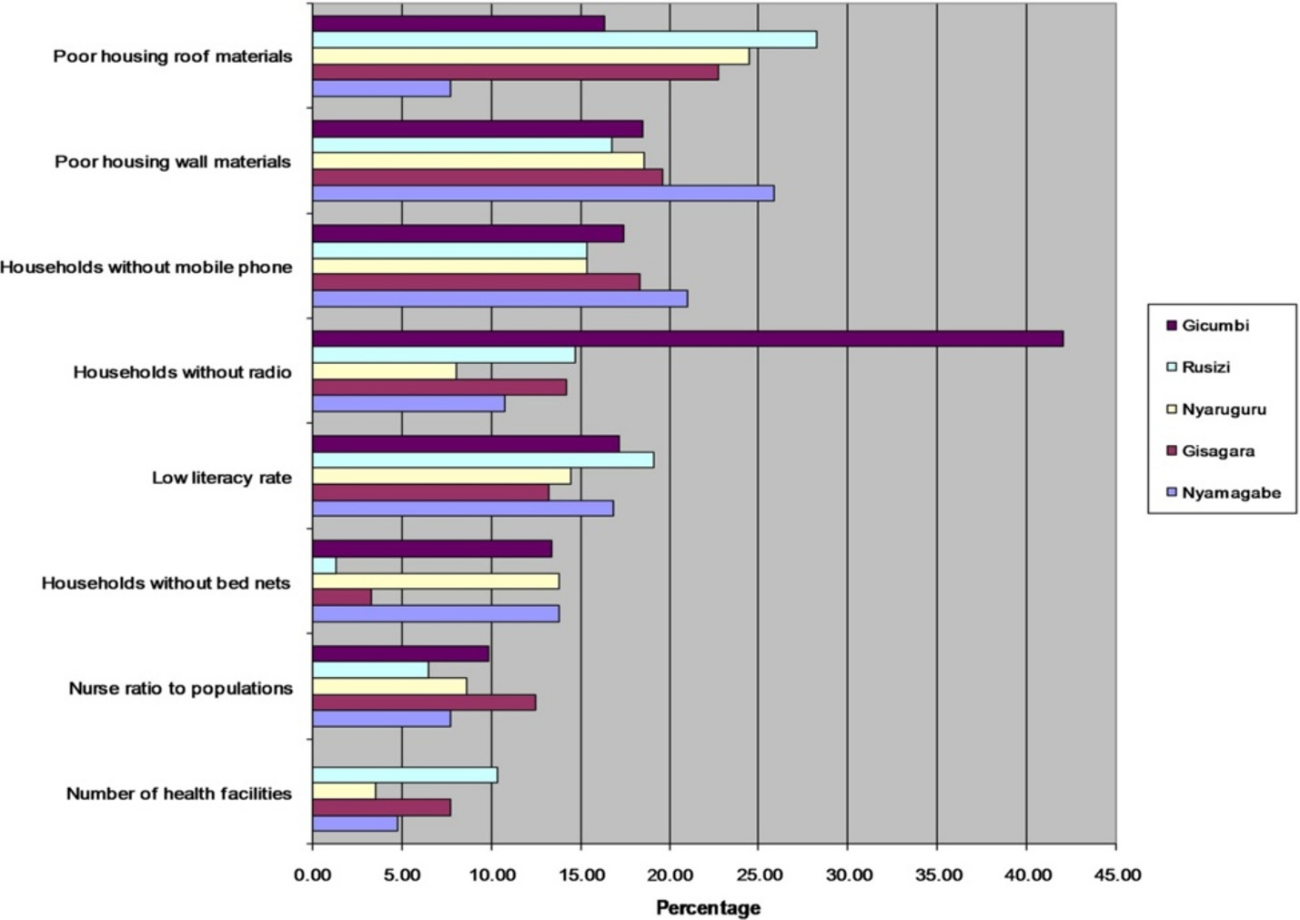
**Less resilient districts and underlying factors.**

The bar charts for the most vulnerable districts in Figure 4 indicate which districts are less resilient, the relative share and contribution of the underlying factors of vulnerability. For lack of resilience domain, the poor housing wall and roof materials and low literacy rate are the main factors that are hampering the capacity of Gicumbi, Ruzizi, Nyaruguru, Gisagara and Nyamagabe districts to anticipate malaria mosquito-biting exposure (Figure 3). The vulnerability of Gicumbi, Nyaruguru and Nyamagabe districts is also exacerbated by the low rate of bed nets ownership low immune highland populations. Because the most vulnerable districts are mostly located in remote rural areas near the borders, access to communication through mobile technology is an impeding factor. With regard to capacity to cope with or to recover from malaria infection, the limited number of health facilities and insufficient medical personnel call for more improved interventions in Gicumbi, Rusizi and Gisagara districts. In these highland districts prone to epidemics malaria induced by climate change and variability, malaria incidence may combine with limited health infrastructure and poverty to result in high morbidity and mortality.

**Figure 5:**
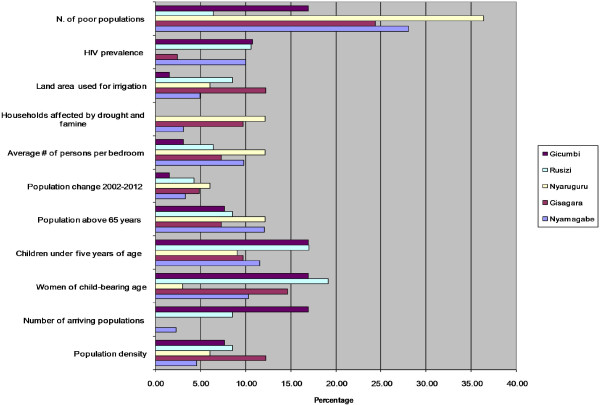
**Most susceptible districts and relative share of underlying factors.**

With regard to susceptibility domain, the district of Gicumbi is the most vulnerable because of poverty, high number children under five years of age, high number of child-bearing age women, and high number of elder populations (Figure 5).

The vulnerability of Gicumbi is also exacerbated by high number of arriving and mobile populations, particularly the refugees from North Kivu in Democratic Republic of Congo who are hosted in Gihembe camp. The vulnerability of Rusizi district is largely associated with a high number of child-bearing age women, high number of children under five years, high population density and high rate of HIV prevalence compared with other districts [86]. The number of arriving populations was also noticed in Rusizi district. The susceptibility of Nyaruguru district is mostly explained by poverty, high number of persons per bedroom, high number households affected by famines, and high number of elder populations. The most influencing indicators in Gisagara district are poverty, high number of child-bearing age women, the irrigated livelihoods that increase the exposure of local population to mosquitoes’ bites, and high population density. The indicators that make Nyamagabe district more susceptible are mainly the poverty and demographic pressure.

### Influence of input indicators on vulnerability index

**Figure 6:**
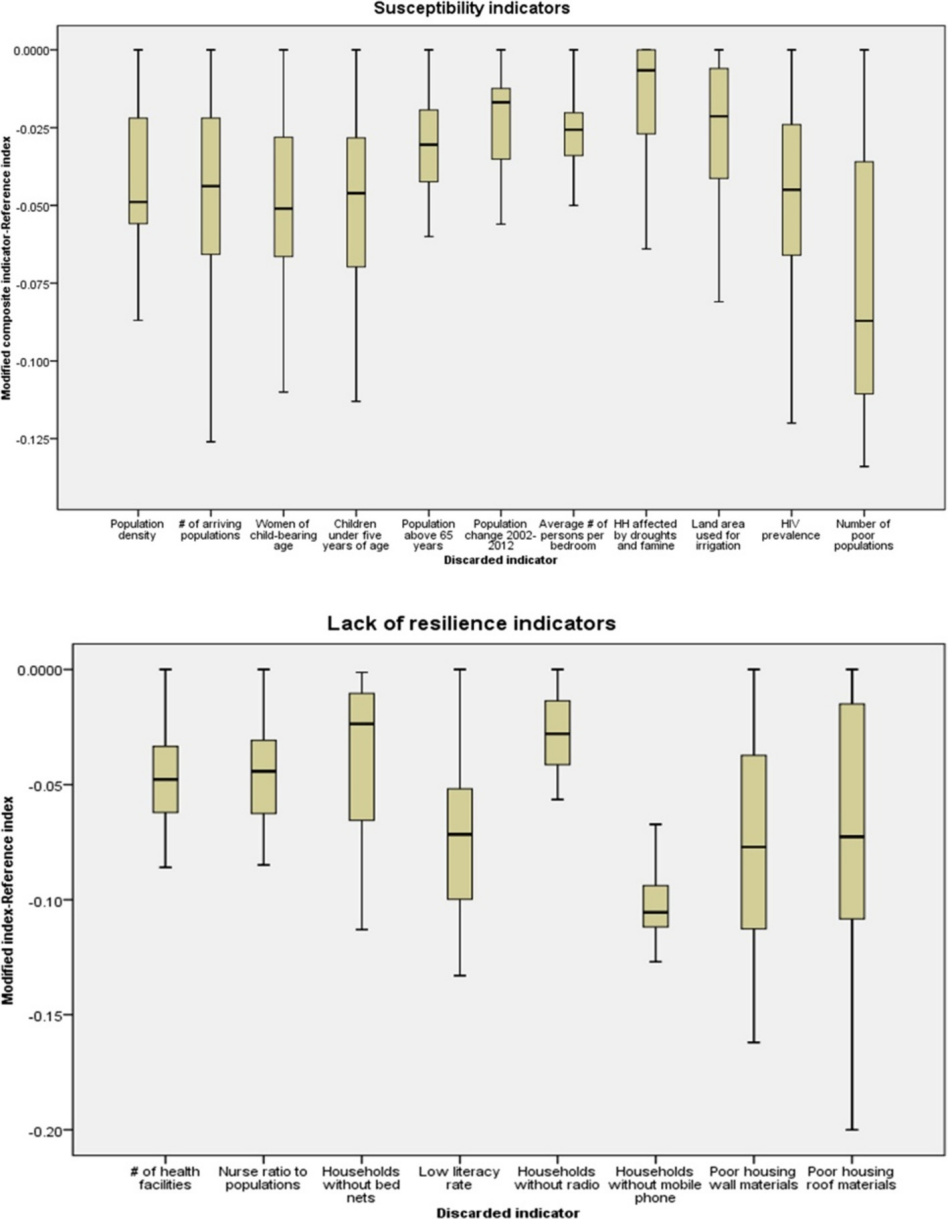
**Box plots showing the influence of indicators on vulnerability index.**

The composition of susceptibility and lack of resilience was evaluated in terms of their influence on final composite indicator of social vulnerability to malaria. The influence of each of the single indicator was calculated by discarding one input at the time while keeping all other all settings remain constant. For susceptibility domain, the results show that the average number of persons per bedroom, elder populations above 65 years, population changes and population density have less impact on final vulnerability index. Conversely, the number of poor populations, number of arriving populations, number of children under five years and women in child-bearing age have an excessive influence on the final composite vulnerability index. In Figure 6, the box plots were used to show the influence of the single indicator on the composite vulnerability index.

For lack of resilience domain, the number of households without mobile phones and the number of households without radio are the less influential indicators. On the other hand, poor housing wall materials, poor housing roof material, number of households without bed nets have a marked impact of vulnerability index.

### Association between the used indicators and malaria incidence

The regression analysis was used to validate the relevance of indicators by highlighting the most sensitive to influence malaria occurrence. Table 6 shows the spearman correlation coefficients between the used indicators and malaria positivity rate for the year 2010.

**Table 6:** **Correlation between social indicators and malaria incidence**

Vulnerability domains	Indicators	R	R^2^	p value
**Susceptibility**	Population density			
	Number of arriving populations	0.057	0.003	0.052
	Women of child-bearing age	−0.401*	0.161	0.766
	Children under five years of age	−0.437*	0.191	0.028
	Population above 65 years	−0.382*	0.146	0.016
	Population change 2002-2012	0.729**	**0.531**	0.037
	Average number of persons per bedroom	0.531**	**0.282**	**0.000**
	Households affected by drought and famine	0.591**	**0.349**	**0.003**
	Number of poor populations	−0.018	0.000	0.494
	Land area used for irrigation	0.611**	**0.373**	0.927
	HIV prevalence in population of 15–49 years	−0.130	0.017	**0.000**
	Number of poor populations	0.018	0.000	0.494
**Lack of resilience**	Number of health facilities	−0.049	0.002	0.796
	Nurse ratio to populations	0.208	0.043	0.796
	Households with bed nets	−0.398*	**0.158**	0.269
	Low literacy rate	0.136	0.018	**0.030**
	Households without radio	0.190	0.036	0.473
	Households without mobile phone	−0.174	0.030	0.314
	Poor housing wall materials	0.378*	**0.143**	**0.040**
	Poor housing roof materials	0.254	0.065	0.040

The most significant indicators are highlighted in bold. The sin *means that the correlation is significant at the 0.05 level (two-tailed), and the sign **shows that the correlation is significant at the 0.01 level (two-tailed). The malaria data used have been collected by Rwandan Ministry of Health at health centre catchment’s area for the year 2010 and then aggregated at district level.

The susceptibility indicators that are scientifically correlated with malaria incidence are highlighted in bold. The population change 2002–2012, land area used for irrigation, households affected by drought and famine and average number of persons per bedroom have the high correlation coefficient values (**r**) raining from 0.729 to 0.531. The susceptibility indicators that have low values of correlation coefficient are the number of poor populations, number of arriving populations and, HIV prevalence and (r value range from 0.018 to 0.130).

This association was expected since the increase in populations augment their contact with an infective mosquito feeding on humans in high transmission areas [87]. Owing to population increase in Rwanda, people moved to the lowlands and –malaria endemic districts such as Nyagatare, Gatsibo and Kayonza [88] with an increasing vulnerability of non-immune migrants. A moderate positive association between the number of persons per bedroom and malaria positivity rate was also found. When household size increases, bed nets are not properly used. Evidence has shown that people sharing a bed net with more than five people are more likely to have malaria than those sharing a bed net with up to two people [89]. The districts with the highest average number of persons per bedroom are Nyagatare, Ngoma and Bugesera. This exacerbates the vulnerability of these most endemic-malaria districts of Rwanda.

A strong relationship between the number of households affected by drought and malaria is not surprising. While prolonged droughts reduce malaria transmission or turn rivers into strings of pools, preferred mosquito breeding sites after short rain, they may also reduce the food security, increase malnutrition and people susceptibility to malaria [90]. The drought-prone districts in Rwanda are Bugesera, Kirehe, Ngoma, Rwamagana, Kayonza, Gatsibo and Nyagatare, Nyanza, Gisagara, Huye and Rusizi [91]. This relationship between malaria and droughts is justified as the drought–prone districts are also malaria-prone areas.

The land area used for irrigation is also strongly correlated with malaria due to the creation of vector breeding sites by irrigation projects [51]. Currently, most of the wetlands in Rwanda are being reclaimed for irrigated crops [92]. A moderate correlation was also found between the number of children under five years and elder populations.

The lack of resilience indicators that have the high correlation coefficient values (r) are poor housing wall materials and the bed nets ownership with the correlation coefficient values of 0.378 and −0.398 respectively. Once again, the positive association between housing conditions and malaria incidence at district level in Rwanda highlights the need for effective malaria intervention that also target the housing improvement in rural areas in addition to bed net provision. Malaria decreases as the number of households with bed nets increases because intense malaria interventions with mass distribution of bed nets led to substantial malaria decline in Rwanda since 2006.

## Discussion

The present work was conducted to assess the social vulnerability to malaria in Rwanda independent of the spatial distribution of the disease based on purely statistical approach and a composite indicator framework. The results from this study are salient for public health policy- and decision makers in malaria control in Rwanda. This spatial assessment is a well-timed support to the national integrated malaria initiative which to seeks to improve the efficacy, effectiveness and sustainability of malaria control interventions through advocacy, social mobilization and inter-sectors collaboration to optimize the allocation of limited resources and health infrastructure at national scale level.

By displaying the lack of resilience versus susceptibility to malaria, the adopted approach provides useful information for decision-makers and a way of communicating the complex interactions between relevant factors of vulnerability to malaria. Squeezing the complex system of social conditions into a single vulnerability index, the developed approach yields a powerful comparative assessment tool capable of capturing societal conditions in a given district that drive people’s vulnerability to malaria infection. This has important policy implications as a successful reduction of malaria burden requires combining the best set of strategies that address the most important vulnerability factors in the most vulnerable districts.

By the fact that most of the vulnerable districts are located in the highlands where unstable malaria transmission would be limited by low temperatures, with the exception of Nyagatare and Bugesera this has an implication for malaria control program in Rwanda. In these low malaria transmission settings, malaria burden is difficult to assess, its impact is significant, and the cost-effectiveness of interventions to predict and respond is doubtful [93]. As an example, malaria epidemics have been reported in Rwanda highlands since 1940s. [94]. Later in the 1980s a steady malaria increase was attributable to low immune population movements from Nyarutovu highlands within Nemba Hospital catchment’s area to the lowlands in the East [95]. Likewise, malaria increased during 1983 to 1987 at Gikonko health centre (Gisagara District), as a result of climate warming near altitude limits of malaria transmission [96]. Unexpected rainfall also resulted in change of malaria patterns in northern Rwanda [95]. In Byumba district hospital catchment’s area in Gicumbi district, a sharp increase of malaria among pregnant women and children was linked to rainfall anomalies in 1998 [97]. It was therefore hypothesized that higher monthly rainfall increased malaria incidence in Byumba highlands, averaging at 2,300 m.

According to Rwanda Ministry of Health, ten border districts have the highest malaria prevalence [98]. The remoteness of rural communities near the borders makes it difficult to provide adequate health services. When border control is inadequate, population’s vulnerability to malaria increases [99]. Near the border areas, malaria infection may not be linked to location at which population came into contact with infective bite(s) of *Anopheles* vectors [100]. Although long-term migrations are limited in Rwanda, temporary migrations with a significant heterogeneity were pointed out by Blumenstock [101], where cross-border movements are responsible for malaria transmission [5]. Consequently, providing adequate health care facilities and appropriate and effective treatment to mobile communities near the borders would be an efficient malaria intervention. Malaria elimination needs to identify those migrant streams in the most vulnerable districts with potential to transport malaria and to target interventions accordingly to prevent potential epidemics malaria [102].

**Figure 7:**
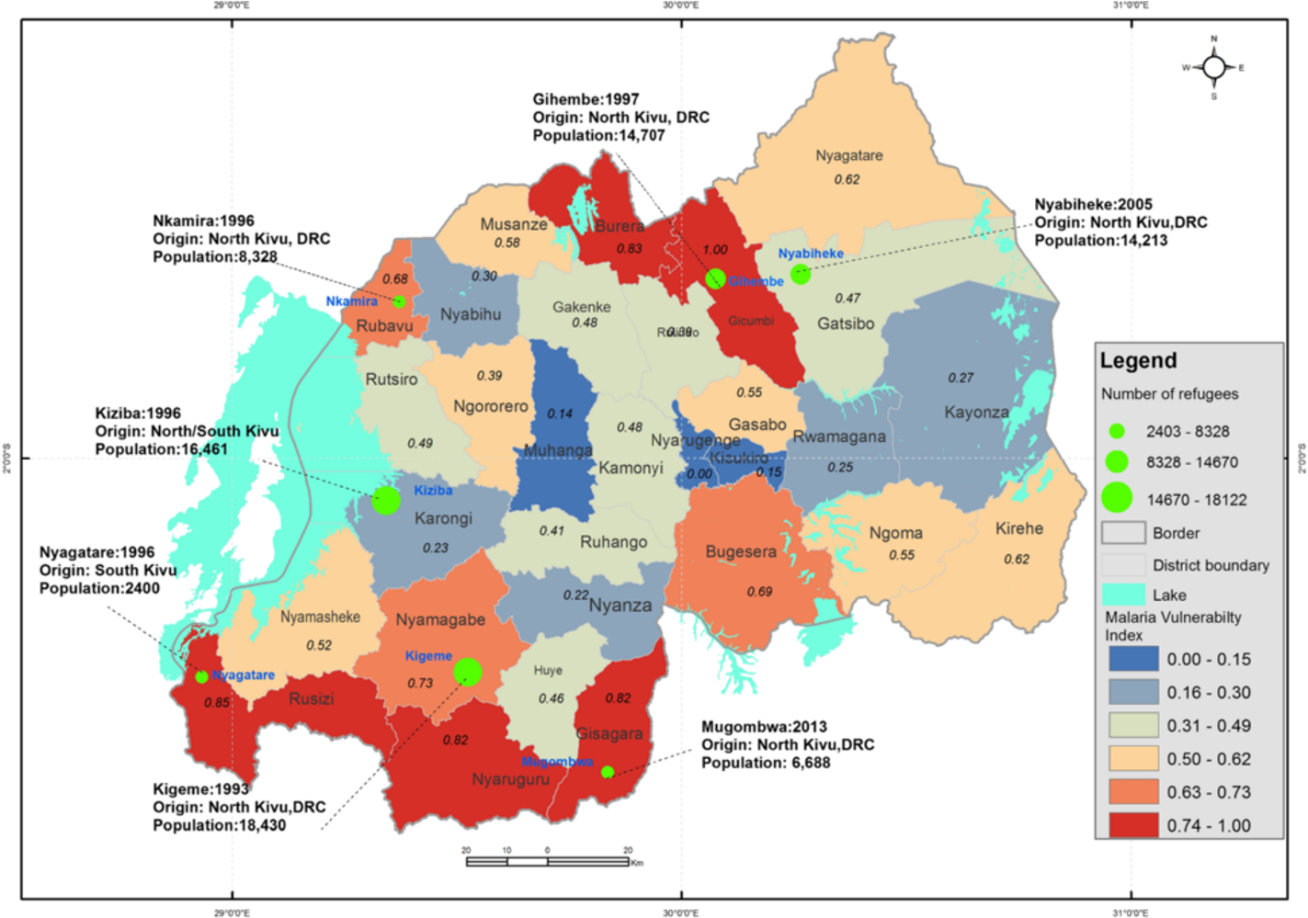
**Malaria vulnerability index and refugees camps.**

Likewise, conflicts are likely to precipitate the movement of refugees across borders and disruption of local infrastructure [99]. They amplify the vulnerability to malaria owing to breakdown of health systems, mass population displacements, and resettlement of refugees in camps in malaria prone areas. Figure 7 shows the location of refugee camps in relation to the levels of social vulnerability to malaria among the district of Rwanda.

In emergency situations, malaria flourishes as a result of breakdown health facilities, displacement of non-immune populations to malaria-prone areas, malnutrition, inappropriate access to treatments, and lack of appropriate shelter [103]. Since 1996, conflicts have resulted in displacement of refugees seeking sanctuary in Rwanda.

Based on recent statistics of the Ministry of Disaster Management and Refugee Affairs in Rwanda, the refugee camps population was estimated to 73,786 until the end of April 2014. The largest camps are Kigeme (18,430), Kiziba (16,461), Gihembe (14,707), Nyabiheke (14,213) and Mugombwa (6,688). As livelihood opportunities are very limited for refugees who generally live in poor housing [104], refugees are more vulnerable to malaria infection. Particular attention should be paid to Mugombwa refugee camp, which is located in the most malaria-endemic area within the most vulnerable district of Gisagara.

Additionally, it is apparent that high population numbers for the most vulnerable age groups (children under five years, women in child-bearing age and elder population) largely explains the susceptibility to malaria in highlands districts. Higher population density and pressure in the highland districts result in stressed productive land as a result of land fragmentation and declining landholding size, pushing people to settle in unsuitable locations or to migrate to malaria-endemic areas with an increasing human exposure to malaria. In past five years, malaria-endemic districts attracted internal migrants are Kayonza, Gatsibo, Rwamagana, Kirehe, and Bugesera in Eastern Province, but also Gasabo and Kicukiro districts within Kigali [105]. The population increase and high average household size may act as impeding factor for effective use of bed nets in Nyagatare and in Nyaruguru districts [88]. This research finding implies that in regions with large households or large populations sharing sleeping room such as refugee camps or worker migration camps near the rice farming areas, the elimination of malaria will require segmenting sleeping quarters into smaller units, such as with mosquito nets.

The low rate of bed nets ownership exacerbates the exiting vulnerability of Gicumbi and Nyaruguru districts. These districts are characterized by low malaria endemicity justifying the low level of malaria interventions with IRS and LLITNs. However, preventive measures targeted to potential hotspots of malaria transmission in highland districts could effectively help in for preventing epidemics and highland malaria. In Nyaruguru, Gisagara and Rusizi District, poverty and poor housing conditions suggest that housing characteristics should be considered in malaria interventions to prevent mosquitoes entering houses and reduce mosquito-biting exposures. This result is in strong agreement with the fourth population and housing census report in Rwanda [106] which revealed that the high percentage of households with poor housing walls in Southern Province is a result of poverty in rural populations. The population increase coupled with land use changes through the extensive marshlands reclamation for irrigation in Gisagara and Nyagatare districts should also attract the attention of public health planners [107].

**Figure 8:**
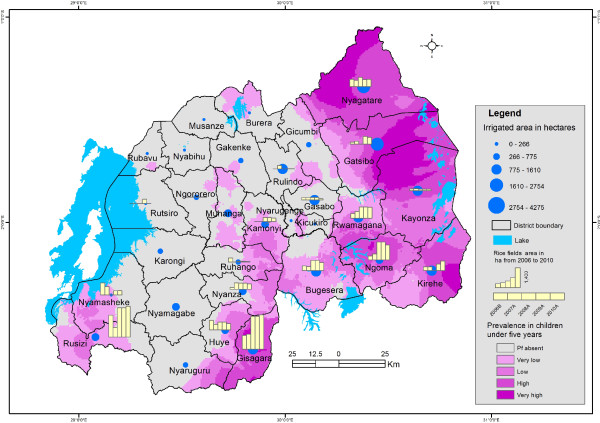
**Land area used for irrigation and malaria parasite prevalence.**

As the area allocated to irrigation is strongly associated with malaria incidence, this has an implication for malaria control program, to take into account the livelihood activities which interplay with the ineffective use and non-use of bed nets to increase the vulnerability to malaria. This is more important in the country like Rwanda where marshlands irrigation as adaptation to climate variability and food shortage has favoured the development of mosquitoes and malaria expansion in highlands [108]. In responding to food shortage, fish ponds in valley bottoms shaped the creation of vector breeding sites [109]. Since 2008, marshland irrigation is being promoted for sustainable food security [110]. Currently, most wetlands in Rwanda are reclaimed for growing rice [93] and rice production increased from 11,949 tons in 2000 to 72,000 tons in 2009 [111]. Rice cropping is a promising solution to food insecurity but also increases malaria in local communities [46] as reported by farmers in Cyabayaga and Rugeramigozi wetlands [92]. Association between malaria and marshlands cleaning is also confirmed by the coincidence of spatial distribution of malaria parasite prevalence rate and area affected by irrigation per district as illustrated in Figure 8.

Ijumba and Lindsay [46] argued that irrigation increases an unstable malaria transmission, where people have little immunity. The vulnerability of irrigation communities may however, be low in some areas owing to better social conditions and farmers’ awareness of bed net use [46]. Planners should consider this opportunity to improve health care facilities when planning marshland irrigation in unstable malaria transmission zones. Integrated malaria control and health impact assessments are recommended near irrigation schemes.

The proposed indicator framework is however without limitations and comes along with some challenges. First, malaria vulnerability assessment based on administrative boundaries may be incomplete for making decisions in complex situations. The composite index based on district administrative boundaries in Rwanda may not be always appropriate for explicitly displaying spatial vulnerability to malaria because boundaries rarely correspond to variations in prevailing social vulnerability at country level [112]. Moreover, the scale is often determined by what is conveniently collected rather than by what is appropriate [113]. Beside, the transmission of malaria does not respect the artificial boundaries which are defined for administrative and political purposes. The PCA weighting scheme does not consider how indicator influence might change across the district. Since PCA analysis is a global statistical analysis, factors and components loadings will not vary at the local scale, and therefore extracted components using the standard PCA do not depend on location. Thus, a strategy for integrating indicators for assessing the social vulnerability to malaria in explicit manner [114] and to examine how the influence of indicators changes across the districts through statistical methods that correct for spatial processes may be required. Secondly, additional indicators such as extent and coverage of IRS campaigns were not available and thus not integrated into the vulnerability analysis because the related data was not available for the entire study area. Once this information is available for the entire country, this could additionally reduce uncertainties in vulnerability. Additionally, some indicators such as social networks and behavior change are difficult to quantitatively measure in malaria vulnerability assessment and therefore not considered in the in this paper. Finally, future research development should focus on an integrated and spatial explicit vulnerability assessment by combining environmental and social drivers is important to achieve an integrated and complete assessment of vulnerability to malaria in order to target malaria interventions that are responsive to the needs of the most vulnerable people in Rwanda.

## Conclusions

This paper applied a composite indicator approach for assessing social vulnerability to malaria among the districts of Rwanda. It is drawn on published works and simplifies the complex information from multisource indicators of vulnerability to malaria into a format that is relevant for decision-making. It shows the most vulnerable districts to integrated social indicators in terms of susceptibility to not withstand malaria and lack of resilience to anticipate, to cope with or to recover from malaria infection. By decomposing vulnerability into its underlying factors, it indicates which factors need to be addressed in each district. The developed composite indicator framework supports the prioritization of appropriate interventions in Gicumbi, Burera, Nyaruguru, Nyagatare, Gisagara, Bugesera, Rusizi, and Burera districts. Being located in the highlands, the prevailing vulnerability in these districts may be exacerbated by cross-border migrations where malaria can be imported from outside.

The health of people in most vulnerable district will not improve unless poverty and expanding inequality are reduced and this includes the effort to control malaria on a large scale. But nothing can be accomplished without positioning the problem in social and cultural contexts of Rwanda. Increasing the community resilience in terms of bed net provision, housing improvement, poverty reduction and access to health care facilities and treatment can be seen as a promising approach for policy makers to be proactive towards malaria in the most vulnerable districts. Besides, improved land-use planning and environmental management can reduce community susceptibility to malaria in Rwandan highlands. Policies enabling activities towards border-crossing populations and enhancement of vector control is needed. As the indicators were aggregated at district level, a study using disaggregated data at household level may be needed. Finally, the approach used provides a comparative assessment and generalizes the relative levels of vulnerability at among the district of Rwanda, but does not provide useful information about what areas within the districts are most vulnerable to malaria infection. A lack of spatial details on prevailing vulnerability within the same district can result in the implementation of uniform interventions that do not necessarily translate to uniform vulnerability reduction inside the district.
